# Hypoxia-inducible factor-2 alpha promotes the proliferation of human placenta-derived mesenchymal stem cells through the MAPK/ERK signaling pathway

**DOI:** 10.1038/srep35489

**Published:** 2016-10-21

**Authors:** Chengxing Zhu, Jiong Yu, Qiaoling Pan, Jinfeng Yang, Guangshu Hao, Yingjie Wang, Lanjuan Li, Hongcui Cao

**Affiliations:** 1 The State Key Laboratory for Diagnosis and Treatment of Infectious Diseases, First Affiliated Hospital, College of Medicine, Zhejiang University; Collaborative Innovation Center for Diagnosis and Treatment of Infectious Diseases, 79 Qingchun Rd., Hangzhou City, 310003, China

## Abstract

Human placenta-derived mesenchymal stem cells (hPMSCs) reside in a physiologically low-oxygen microenvironment. Hypoxia influences a variety of stem cell cellular activities, frequently involving hypoxia-inducible factor-2 alpha (HIF-2α). This research showed that hPMSCs cultured in hypoxic conditions (5% O_2_) exhibited a more naïve morphology and had a higher proliferative capability and higher HIF-2α expression than hPMSCs cultured in normoxic conditions (21% O_2_). Similar to the hypoxic cultures, hPMSCs over-expressing HIF-2α showed higher proliferative potential and higher expression of CCND1 (CyclinD1), MYC (c-Myc), POU5F1 (Oct4) and the components of the MAPK/ERK pathway. In contrast, these genes were down-regulated in the HIF-2α-silenced hPMSCs. After adding the MAPK/ERK inhibitor PD0325901, cell growth and the expression of CCND1 and MYC were inhibited. Furthermore, the chromatin immunoprecipitation (ChIP) assay and electrophoretic mobility shift assay (EMSA) showed that HIF-2α bound to the MAPK3 (ERK1) promoter, indicative of its direct regulation of MAPK/ERK components at the transcriptional level during hPMSC expansion. Taken together, our results suggest that HIF-2α facilitated the preservation of hPMSC stemness and promoted their proliferation by regulating CCND1 and MYC through the MAPK/ERK signaling pathway.

Mesenchymal stem cells (MSCs) are one of the most promising candidates for regenerative medicine and clinical therapeutic applications because of their multipotent characteristics^1^,^2^. These cells possess the properties of adult stem cells, which are able to regenerate spontaneously and differentiate into various types of progenitor cells^3^. Among the diverse tissues that contain MSCs, human placenta-derived mesenchymal stem cells (hPMSCs) are a good candidate for clinical applications to treat diseases such as hepatopathy^4^,^5^ because they are prevalent and have low immunogenicity and high differentiation and proliferation potential. Nevertheless, these advantages may be restricted by inappropriate cultivation and the influence of environment changes *in vitro*, leading to poor outcomes in hPMSC transplantation for clinical therapeutic applications. Therefore, it is important to explore better methods to maintain the benefits of hPMSCs *in vitro*.

The placenta and fetus reside in a hypoxic microenvironment (1–5% O_2_) *in vivo*^6^; therefore, hPMSCs also reside in such an environment. Notably, numerous articles have reported that hypoxia induces cellular stress responses in many cell types. For example, cancer cells and stem cells modify a variety of functions under low oxygen tension^7^,^8^,^9^,^10^. In particular, hypoxia was shown to facilitate cell growth and survival. Although various functions of hypoxia have been revealed, the role of hypoxia in hPMSCs is unclear. Because low oxygen culture conditions mimic the stem cell niche, an understanding of the associated molecular mechanisms may help preserve the properties of hPMSCs. Hypoxia-inducible factor-2 alpha (HIF-2α) is an important effector in low oxygen tension, which induces the transcription of target genes relevant to the hypoxic stress response. Therefore, some fundamental vital cellular activities are impacted by HIF-2α (EPAS1) in hypoxia, such as cell migration, survival, differentiation, apoptosis and metabolism^11^,^12^,^13^,^14^. The primitive cellular state is one of the significant advantages of MSCs; the more primitive the MSCs, the more properties of stem cells they retain, such as a high self-renewal potential. Proliferation is the fundamental activity of stem cells, and the proliferative potential impacts hPMSC transplantation in clinical regenerative and therapeutic applications. Therefore, we questioned whether HIF-2α also helps maintain the favorable stem cell properties of hPMSCs that remain in a hypoxic niche. If so, what is the mechanism involved in regulating HIF-2α in hPMSCs?

A wide variety of signal transduction pathways participate in fundamental cellular activities, and they may also play a role in the cellular response to changes in the external environment. Certain cellular stress conditions, such as inflammation, reactive species, and mitogens, frequently activate the mitogen-activated protein kinase (MAPK) pathway, which is highly conserved in metazoans and controls many principal processes involved in cell metabolism, motility, proliferation, differentiation and apoptosis^15^,^16^,^17^,^18^. Based on previous research, four MAPK signal transduction pathways have been reported: the ERK, p38 MAPK, JNK/SAPK, and ERK5/BMK1 signaling pathways. The MAPK/ERK pathway, also known as the Ras-Raf-MEK-ERK pathway phosphorylates a spectrum of substrates (including phosphatases, kinases, cytoskeletal proteins and transcription factors) and plays an integral role in cell cycle progression and survival^19^,^20^,^21^,^22^. ERK1/2 are the downstream components of the MAPK/ERK pathway and mediate cell expansion processes after transcriptional activation by certain factors^23^,^24^,^25^. Hypoxia is a common stress in physiological environments and pathological conditions, and HIF-2α may activate the MAPK/ERK pathway and interact with its main signaling molecules to influence hPMSC proliferation.

This study aimed to explore the function of HIF-2α in hPMSCs, which might help explain the influence of hypoxia on hPMSC activities. Furthermore, the signal transduction mechanisms involved in regulating HIF-2α and the interactions of HIF-2α and relevant genes were investigated in detail. This research is important for future clinical applications and basic scientific research of hPMSCs to determine how to preserve and promote the stem cell characteristics of hPMSCs.

## Results

### Comparisons of the general biological characteristics of hPMSCs cultured under normoxic and hypoxic conditions

hPMSCs that were cultured in a standard normoxic atmosphere (21% O_2_) grew into typical, adherent monolayer cells with a fibroblast-like appearance. However, hPMSCs that were grown in a hypoxic atmosphere (5% O_2_) became comparatively smaller in size, with a naive spindle-shaped morphology (see Supplementary Figure S1A), which represented a more primitive stem cell state. The 5-ethynyl-2′-deoxyuridine (EdU) flow cytometry cell proliferation assays showed that the proliferation rates of hPMSCs that were cultured in a hypoxic atmosphere for 4 hours were obviously increased compared with the cells that were cultured in a normoxic atmosphere (see Supplementary Figure S1B). Additionally, the relative protein and mRNA expression levels of EPAS1 (HIF-2α) were elevated in the low-oxygen group compared with the atmospheric air group (see Supplementary Figure S1C). Taken together, these results suggested that a physiologically hypoxic atmosphere (5% O_2_) favored a more primitive morphology, higher hPMSC proliferation rates, and increased HIF-2α expression.

### HIF-2α positively regulates hPMSC proliferation

**Figure 1:**
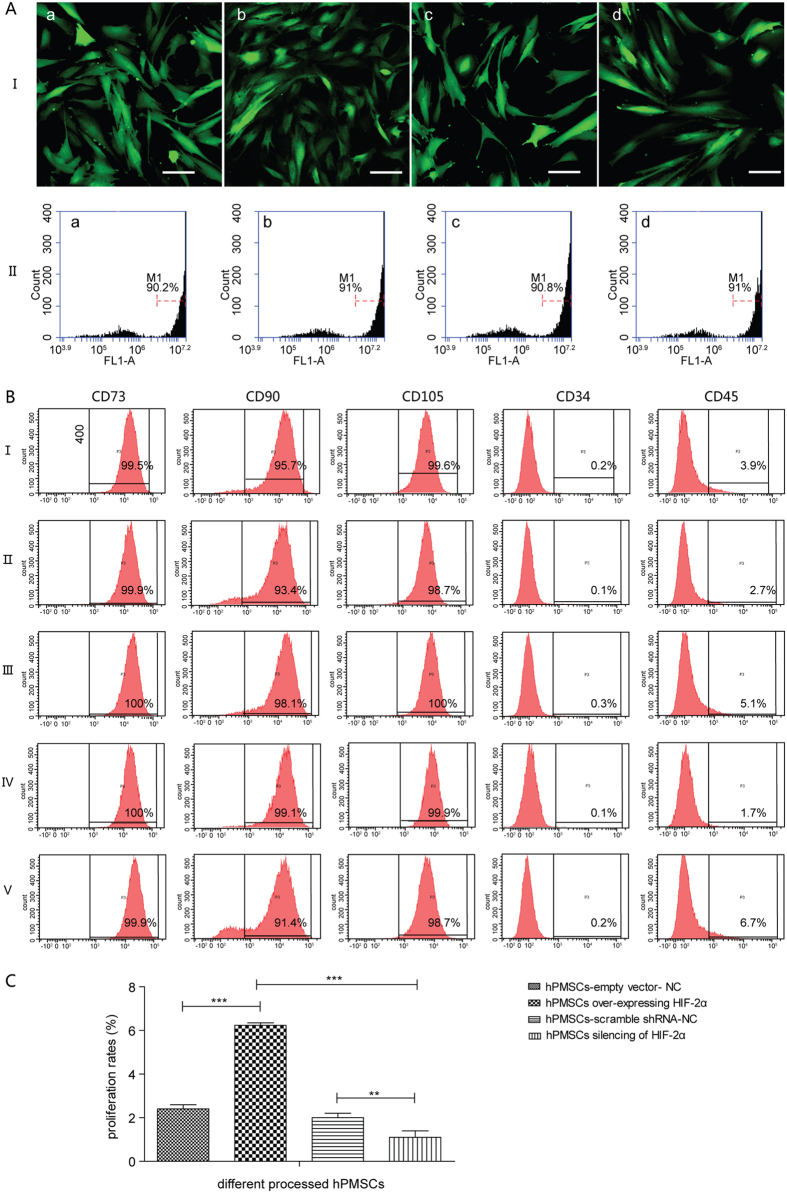
Comparisons of the four types of processed hPMSCs to explore the functions of HIF-2α. (**A**) (I): Morphologies of the four groups of processed hPMSCs using confocal microscopy (20×, scale bars, 50 μm). (**A**) (II): GFP expression in the four groups of processed hPMSCs, as assessed by the FACS assay. (a) hPMSCs-empty vector-NC, (b) hPMSCs over-expressing HIF-2α, (c) hPMSCs-scramble shRNA-NC, and (d) HIF-2α-silenced hPMSCs. (**B**) Analysis of the surface antigens (CD73, CD90, CD105, CD34, and CD45) on hPMSCs from the different treatment conditions: (I) untreated control hPMSCs, (II) hPMSCs-empty vector-NC, (III) hPMSCs over-expressing HIF-2α, (IV) hPMSCs-scramble shRNA-NC, and (V) HIF-2α-silenced hPMSCs. (**C**) Statistical graph of the comparisons of the cell proliferation rates of the different processed hPMSCs determined using EdU flow cytometry. The tests were performed in triplicate and repeated in three independent experiments. The data are presented as the mean ± S.D. (error bars) and were statistically analyzed using one-way ANOVA. **p < *0*.*01*, ***p < *0*.*001*.

**Figure 2:**
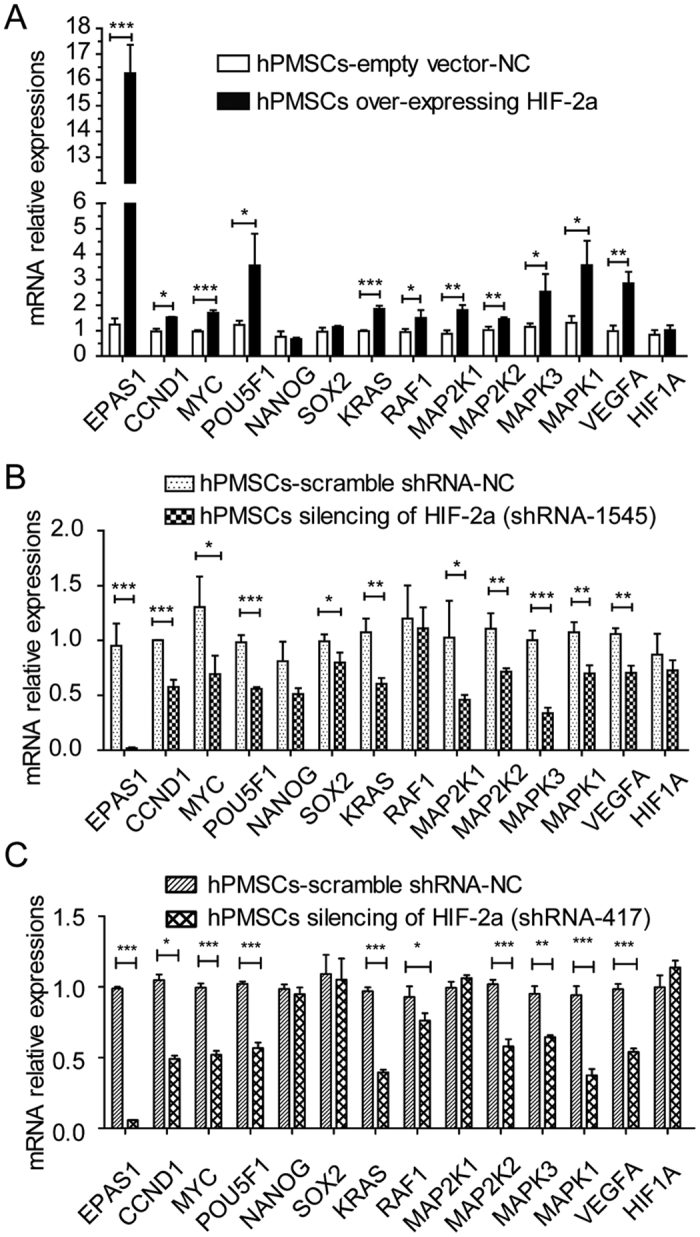
Relative mRNA expression levels of specific genes in the different groups of processed hPMSCs. (**A**) Quantitative real-time polymerase chain reaction (qRT-PCR) results from hPMSCs over-expressing HIF-2α compared with the hPMSCs-empty vector-NC. The tests were performed in triplicate and repeated in three independent experiments. The data are presented as the mean ± S.D. (error bars) and were statistically analyzed using Student’s t-test. *p < *0*.05, **p < *0*.01, ***p < *0*.*001*. (**B**,**C**) The qRT-PCR results from the HIF-2α-silenced hPMSCs (**B**: shRNAs 1545, **C**: shRNAs 417) compared with the hPMSCs-scramble shRNA-NC. The tests were performed in triplicate and repeated in three independent experiments. The data are presented as the mean ± S.D. (error bars) and were statistically analyzed using Student’s t-test. *p < *0*.*05*, **p < *0*.*01*, ***p < *0*.*001*.

**Figure 3:**
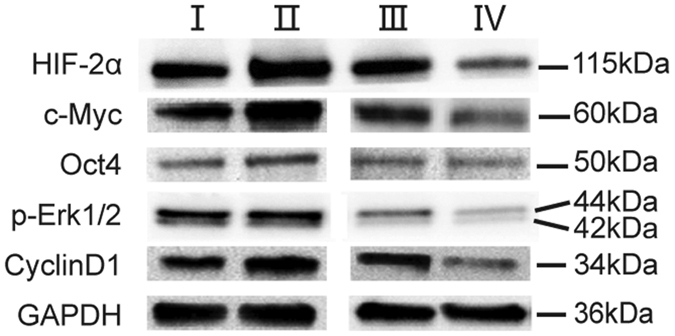
Western blots (WBs) of HIF-2α, c-Myc, Oct4, p-Erk1/2, and CyclinD1 in the four groups of processed hPMSCs. (I) hPMSCs-empty vector-NC, (II) hPMSCs over-expressing HIF-2α, (III) hPMSCs-scramble shRNA-NC, and (IV) HIF-2α-silenced hPMSCs (shRNA1545). The original immunoblots were presented in Supplementary Figure S3.

**Figure 4:**
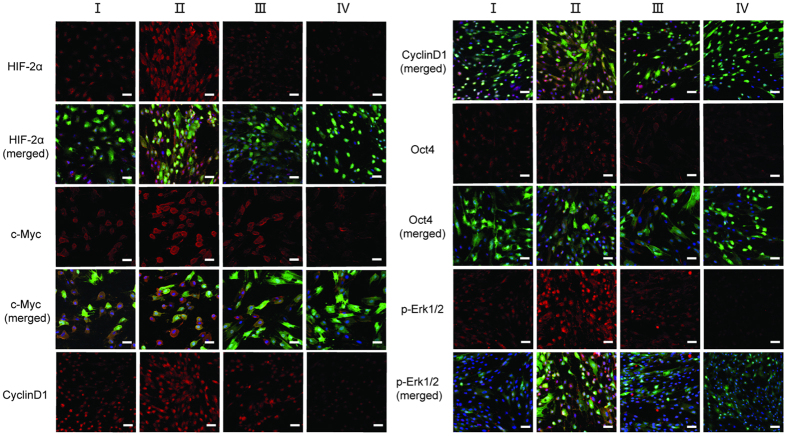
Immunofluorescence microscopy (IF) of HIF-2α, c-Myc, CyclinD1,Oct4, and p-Erk1/2 in the four groups of processed hPMSCs (20×, scale bars, 50 μm). (I) hPMSCs-empty vector-NC, (II) hPMSCs over-expressing HIF-2α, (III) hPMSCs-scramble shRNA-NC, and (IV) HIF-2α-silenced hPMSCs (shRNA1545).

hPMSCs over-expressing HIF-2α and the corresponding negative control [hPMSCs with an empty vector (hPMSCs-empty vector-NC)] were studied to further investigate the effect of HIF-2α. In the opposite experiment, hPMSCs in which HIF-2α was silenced with shRNA and a negative control [hPMSCs with a scrambled shRNA (hPMSCs-scramble shRNA-NC)] were used. We first tested for successfully infected hPMSCs using confocal microscopy and a FACS assay, which showed a constant morphology and green fluorescent protein (GFP) expression in these four different treatment groups (Fig. 1A). These four groups of reconstructed hPMSCs shared a similar expression of typical surface antigens with untreated hPMSCs, which were positive for CD73, CD90, and CD105 and negative for CD34 and CD45 (Fig. 1B). The groups maintained the representative markers of hPMSCs and the capacity to differentiate into osteoblasts and adipocytes (data not shown). In the EdU flow cytometry assays, the hPMSCs over-expressing HIF-2α showed higher proliferation rates than hPMSCs-empty vector-NC. Conversely, the HIF-2α-silenced hPMSCs showed a reduced proliferative capacity compared with hPMSCs-scramble shRNA-NC (Fig. 1C). The proliferation rates of hPMSCs corresponded to HIF-2α expression. Furthermore, HIF-2α expression was higher in the HIF-2α-over-expressing hPMSCs and lower in hPMSCs lacking HIF-2α than in their respective control groups, as indicated by the quantitative real-time polymerase chain reaction (qRT-PCR; Fig. 2), western blots (WBs; Fig. 3) and immunofluorescence staining (IF; Fig. 4). These results showed that HIF-2α positively regulated hPMSC proliferation.

### HIF-2α controls and facilitates the expression of genes involved in hPMSC proliferation and stemness

The relative mRNA expression levels of CCND1 (CyclinD1) and MYC (c-Myc), proliferation-related transcription factors, were increased in hPMSCs over-expressing HIF-2α compared with the hPMSCs-empty vector-NC. Furthermore, the major markers of primitive stem cells, POU5F1 (Oct4), NANOG and SOX2^26^, were also evaluated. POU5F1 expression was increased in hPMSCs over-expressing HIF-2α compared with the hPMSCs-empty vector-NC, but NANOG and SOX2 expression remained unchanged in the two groups (Fig. 2A). In contrast, the HIF-2α-silenced hPMSCs (shRNA1545 and shRNA 417) expressed less CCND1, MYC, POU5F1 and SOX2 than hPMSCs-scramble shRNA-NC, but NANOG expression was not different between the two groups (Fig. 2B,C). These data confirmed that HIF-2α contributed to increase the mRNA expression of CCND1, MYC and POU5F1, which indicated that HIF-2α expression increased hPMSC proliferation and stemness.

### Western blots and immunofluorescence staining verify the qRT-PCR results

The WB results showed that hPMSCs over-expressing HIF-2α expressed higher levels of the HIF-2α, CyclinD1, c-Myc, and Oct4 proteins than the hPMSCs-empty vector-NC. However, their expression was reduced in the HIF-2α-silenced hPMSCs compared with the hPMSCs-scramble shRNA-NC (Fig. 3). Similarly, in the IF staining assays, the expression of HIF-2α, CyclinD1, c-Myc, and Oct4 in hPMSCs over-expressing HIF-2α was increased compared with the hPMSCs-empty vector-NC, but decreased in the HIF-2α-silenced hPMSCs compared with the hPMSCs-scramble shRNA-NC (Fig. 4). Generally speaking, the main qRT-PCR results were confirmed by WB and IF. The results proved that HIF-2α regulated the expression of vital proteins, proliferation markers (CyclinD1 and c-Myc) and markers of stem cells (Oct4), by which HIF-2α expression promoted hPMSC proliferation and stemness.

### HIF-2α regulates hPMSC proliferation through the MAPK/ERK pathway

Based on the qRT-PCR results, the essential components of the MAPK/ERK pathway, KRAS, RAF1, MAP2K1 (Mek1), MAP2K2 (Mek2), MAPK3 (Erk1) and MAPK1 (Erk2), were elevated in hPMSCs over-expressing HIF-2α compared with the hPMSCs-empty vector-NC (Fig. 2A). In contrast, these mRNAs all remained at lower levels in the HIF-2α-silenced hPMSCs (shRNA1545 and shRNA 417) than in the hPMSCs-scramble shRNA-NC, with the exception of RAF1, which did not show a significant difference (Fig. 2B,C). The relative mRNA expression levels of vascular endothelial growth factor-A (VEGFA), the target gene of the hypoxia-inducible factor family, and HIF-1α (HIF1A), an isoform of HIF-2α, were also evaluated to further show that the regulation was mediated by HIF-2α. Compared with the corresponding negative control groups, the expression of VEGFA was increased in hPMSCs over-expressing HIF-2α and decreased in the HIF-2α-silenced hPMSCs. However, HIF1A expression remained unchanged in each group (Fig. 2). Therefore, the important components of the Ras-Raf-MEK-ERK signaling pathway changed along with the expression of HIF-2α at the genetic level. WB and IF verified that the levels of the downstream effectors of MAPK/ERK pathway, phosphorylated Erk1 and Erk2 (p-Erk1/2), were increased in hPMSCs over-expressing HIF-2α compared with the hPMSCs-empty vector-NC, but were maintained at lower level in the HIF-2α-silenced hPMSCs than in the hPMSCs-scramble shRNA-NC (Figs 3 and 4). Taken together, the results indicated that the HIF-2α-mediated regulation of hPMSCs was intimately associated with the MAPK/ERK pathway.

**Figure 5:**
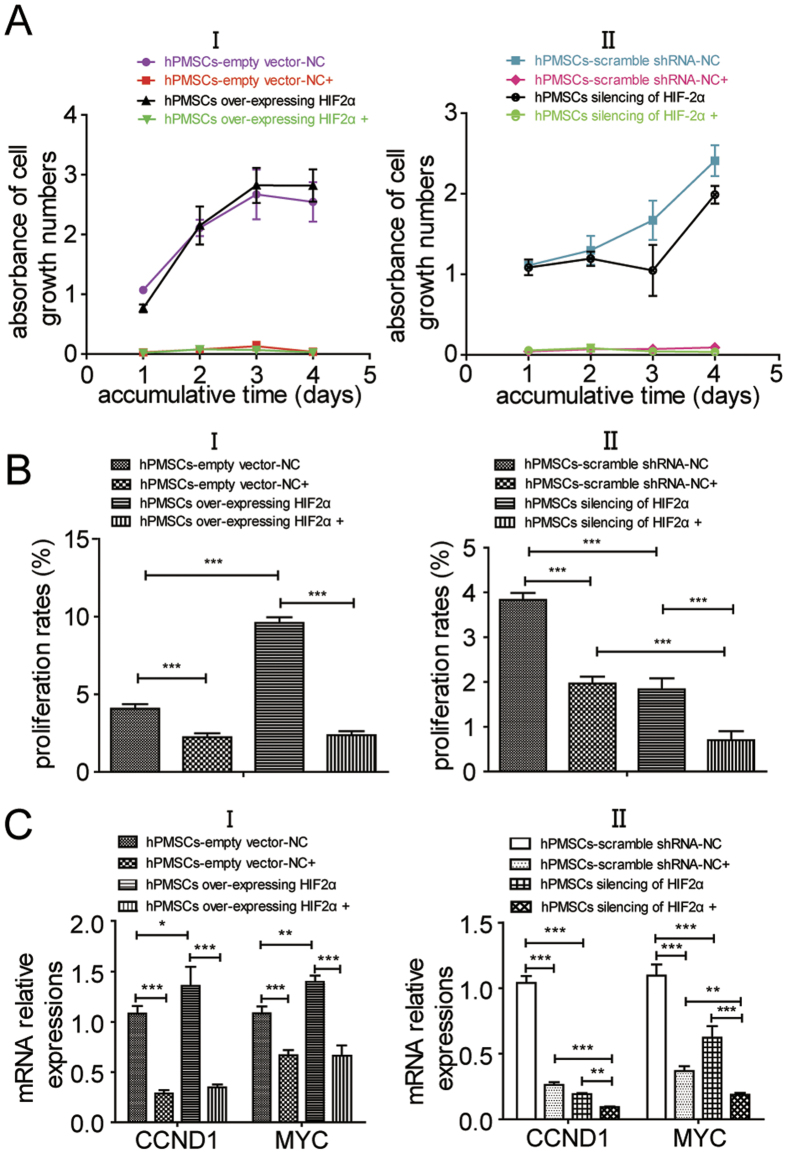
The inhibitor (PD0325901) of the MAPK/ERK pathway blocked hPMSC proliferation, which was facilitated by HIF-2α. (**A**) The results of the CCK-8 assay of the processed hPMSCs in the PD0325901- or DMSO-treated groups after (I) comparisons of the cumulative cell growth between hPMSCs over-expressing HIF-2α and hPMSCs-empty vector-NC in the inhibitor- or vehicle-treated groups, and (II) comparisons of the cumulative cell growth between the HIF-2α-silenced hPMSCs (shRNA1545) and hPMSCs-scramble shRNA-NC in the inhibitor- or vehicle-treated groups. The PD0325901 (inhibitor) treatment was marked as +, but the DMSO (vehicle) treatment was not marked. The tests were performed in triplicate and repeated in three independent experiments. The data are presented as the mean ± S.D. (error bars). (**B**) EdU flow cytometry cell proliferation assays of the hPMSCs that were treated with either PD0325901 or DMSO for 2 days. (I) comparisons of proliferation rates between hPMSCs over-expressing HIF-2α and hPMSCs-empty vector-NC in inhibitor- or vehicle-treated groups, and (II) comparisons of proliferation rates between hPMSCs silencing of HIF-2α (shRNA-1545) and hPMSCs-scramble shRNA-NC in inhibitor- or vehicle-treated groups. PD0325901-treated groups are marked with “+”; DMSO-treated groups are not marked. Tests were performed in triplicate and repeated in three independent experiments. Data results were presented as the mean ± S.D. (error bar) and were statistically analyzed by one-way ANOVA. ***p < *0*.*001*. (**C**) The qRT-PCR results of the processed hPMSCs in the PD0325901- or DMSO-treated groups; (I) comparisons of CCND1 and MYC expression between the hPMSCs over-expressing HIF-2α and the hPMSCs-empty vector-NC in the inhibitor- or vehicle-treated groups, and (II) comparisons of CCND1 and MYC expression between the HIF-2α-silenced hPMSCs (shRNA1545) and hPMSCs-scramble shRNA-NC in the inhibitor- or vehicle-treated groups. The PD0325901 (inhibitor) treatment was marked as +, but the DMSO (vehicle) treatment was not marked. The tests were performed in triplicate and repeated in three independent experiments. The data are presented as the mean ± S.D. (error bars) and were statistically analyzed using one-way ANOVA. *p < *0*.*05*, **p < *0*.*01*, ***p < *0*.*001*.

We applied the MAPK/ERK pathway inhibitor PD0325901 to explore the role of signal transduction in HIF-2α-mediated regulation from the reverse side. The CCK-8 assay was used to measure the increasing numbers of viable cells for 5 consecutive days under different conditions (inhibitor or vehicle). After adding PD0325901, the proliferation of hPMSCs over-expressing HIF-2α and hPMSCs-empty vector-NC decreased compared with the corresponding vehicle-treated groups (Fig. 5AI). Similarly, the growth of the HIF-2α-silenced hPMSCs (shRNA1545) and hPMSCs-empty vector-NC was also significantly inhibited by PD0325901 (Fig. 5AII).

Moreover, EdU flow cytometry assays were used to measure hPMSC proliferation 2 days after treatment (PD0325901-treated groups and DMSO-treated groups). The PD0325901-treated groups show a significant decrease in the cell proliferation rate. In the vehicle-treated groups, the proliferation rate of hPMSCs over-expressing HIF-2α was increased compared with the hPMSCs-empty vector-NC. In the inhibitor-treated groups, the differences in the proliferation rates between the hPMSCs over-expressing HIF-2α and the hPMSCs-empty vector-NC were insignificant, indicating that the positive HIF-2α-mediated regulation of hPMSC proliferation was inhibited when the MAPK/ERK pathway was blocked (Fig. 5BI). In the vehicle-treated groups, the proliferation rate of the HIF-2α-silenced hPMSCs was reduced compared with the hPMSCs-empty vector-NC. In the inhibitor-treated groups, the HIF-2α-silenced hPMSCs showed a more significant decrease in the proliferation rate, indicating that HIF-2α silencing and inhibition of the MAPK/ERK pathway had a combined effect of decreasing the proliferation rate of hPMSCs (Fig. 5BII).

Therefore, hPMSC proliferation was inhibited by blocking the MAPK/ERK pathway, indicating that the MAPK/ERK pathway was related to hPMSC proliferation. The effect of HIF-2α expression on hPMSC proliferation was also blocked by PD0325901.

Compared with respective vehicle-treated groups, the relative expression levels of the CCND1 and MYC levels declined in all PD0325901-treated groups after 2 days of treatment, indicating that the expression of proliferation-related genes was reduced when the MAPK/ERK pathway was inhibited. Specifically, in the vehicle-treated groups, the relative expression levels of the CCND1 and MYC mRNAs were increased in the hPMSCs over-expressing HIF-2α compared with the hPMSCs-empty vector-NC, which also verified the result shown in Fig. 2A in which HIF-2α facilitated the expression of proliferation markers. However, in the inhibitor-treated groups, the differences in CCND1 and MYC expression between the hPMSCs over-expressing HIF-2α and the hPMSCs-empty vector-NC were insignificant. Therefore, the HIF-2α-induced increase in the expression of hPMSC proliferation-related genes was prevented by blocking the MAPK/ERK pathway (Fig. 5CI). In contrast, the relative expression levels of the CCND1 and MYC mRNAs were reduced in the HIF-2α-silenced hPMSCs compared with the hPMSCs-scramble shRNA-NC in the vehicle-treated groups, which also verified the result shown in Fig. 2B,C in which the expression of proliferation-related genes was compromised when HIF-2α expression was reduced. In the inhibitor-treated groups, the relative expression levels of the CCND1 and MYC mRNAs in the HIF-2α-silenced hPMSCs were reduced compared with the hPMSCs-scramble shRNA-NC (Fig. 5CII). Therefore, the “dual suppression” from HIF-2α silencing and the inhibitor of MAPK/ERK pathway obviously prevented the expression of proliferation-related genes in hPMSCs. These results confirmed that HIF-2α-induced promotion of hPMSC proliferation was prevented by inhibiting the MAPK/ERK pathway. Taken together, these results indicated that HIF-2α promoted hPMSC proliferation through the MAPK/ERK pathway.

### HIF-2α regulates the transcription of the MAPK3 (Erk1) gene

**Figure 6:**
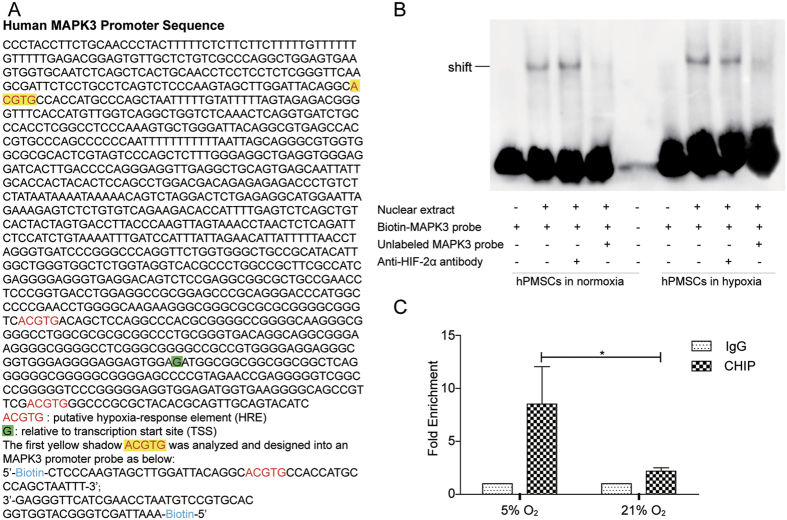
HIF-2α regulates the transcription of the MAPK3 gene, as determined by EMSA and ChIP-qPCR. (**A**) Human MAPK3 promoter sequence. The “ACGTG” sequence is the putative hypoxia-response element (HRE), which may be the candidate HIF-2α binding site. The green shadow “G” site is shown relative to the transcription start site (TSS). The MAPK3 promoter probe was designed with the “ACGTG” sequence. (**B**) Electrophoretic mobility shift assay (EMSA) of the MAPK3 promoter probes and nuclear protein extracts. The lane to which only the biotin-labeled MAPK3 probe was added was the negative control. The anti-HIF-2α antibody was added for the supershift assay. The unlabeled MAPK3 probe was added as a competitor for the biotin-labeled probe. (**C**) ChIP-qPCR detection of HIF-2α binding to the MAPK3 promoter using normoxic and hypoxic hPMSCs. The tests were performed in triplicate and repeated in three independent experiments. The data are presented as the mean ± S.D. and were statistically analyzed using Student’s t-test. *p < *0*.*05*.

This study investigated whether HIF-2α regulated MAPK3 (Erk1) transcription by binding directly to its promoter region and determined whether hypoxia-induced HIF-2α expression promoted hPMSC proliferation through a direct interaction of HIF-2α with the effective signaling molecules in the MAPK/ERK pathway. After analyzing the MAPK3 promoter sequence relative to its transcription start site (TSS), we found three putative hypoxia-response elements (HREs) – ACGTG – that were potential HIF binding sites (Fig. 6A). One potential HIF-2α binding site (yellow highlighting) was selected to synthesize the MAPK3 promoter probe, which was incubated with the hPMSC (cultured in normoxia or hypoxia) nuclear extracts and then analyzed by electrophoretic mobility shift assay (EMSA). The results of the EMSA (Fig. 6B) showed that more HIF-2α from the nuclear protein extracts of the hypoxic hPMSCs bound to the MAPK3 promoter probe than HIF-2α from the normoxic group (shift).

A 200-fold molar excess of unlabeled MAPK3 probe competed for binding with the biotin-labeled MAPK3 probes, as shown by the disappearance of these shifted bands. As expected, adding the biotin-labeled MAPK3 probe alone (negative control) did not produce any bands, with the exception of the bands representing the free probes. Therefore, it is likely that hypoxia induced HIF-2α to bind to the MAPK3 promoter and thereby directly regulated the transcription of the MAPK3 (Erk1) gene in hPMSCs. This result provided further support for the hypothesis that HIF-2α, a hypoxia-induced transcription factor, promotes hPMSC proliferation through the MAPK/ERK pathway.

We conducted the ChIP analysis with hPMSCs grown in hypoxic and normoxic atmospheres to determine intracellular binding and transcription. The ChIP-qPCR assay showed that HIF-2α directly bound to the MAPK3 promoter compared with IgG, and the binding was significantly increased in the 5% oxygen environment (Fig. 6C).

## Discussion

Hypoxia influences a variety of cellular processes, such as proliferation, differentiation, apoptosis, migration, and development^6^,^27^,^28^, in which HIF may play an important function^8^,^29^,^30^. hPMSCs reside in a hypoxic microenvironment *in vivo*. This study examined how hypoxia influenced the characteristics of hPMSCs. Similar to the results reported in previous studies, a physiologically relevant microenvironment of 5% O_2_ promoted high proliferation rates and the naïve morphology of hPMSCs and increased the expression of important hypoxic molecules, such as EPAS1 (HIF-2α), compared with conventional cultures at 21% O_2_. HIF-2α is more stable and maintains significant long-term effects under hypoxic conditions^12^. Proliferation and naïve features are the fundamental properties of MSCs and are important for their clinical application. Therefore, we questioned whether HIF-2α influenced these characteristics of hPMSCs. Furthermore, we paid particular attention to the molecular mechanisms associated with HIF-2α.

We increased or reduced HIF-2α expression to determine its influence on hPMSCs. Using recombinant lentivirus infections, four groups of hPMSCs were established: hPMSCs over-expressing HIF-2α and its negative control, hPMSCs-empty vector-NC, as well as HIF-2α-silenced hPMSCs and its control, hPMSCs-scramble shRNA-NC. The hPMSCs over-expressing HIF-2α showed the highest proliferation rates among the groups, but the HIF-2α-silenced hPMSCs divided slowly, indicating that HIF-2α facilitated the increased proliferation of hPMSCs. Molecular methods were employed to examine the mechanism underlying this result and indicated that HIF-2α over-expression increased the transcription of the proliferation-related factors CCND1 (CyclinD1) and MYC (c-Myc) and increased the transcription of an important naïve stem cell marker, POU5F1 (Oct4). The expression of these genes was significantly decreased in the HIF-2α-silenced hPMSCs. These results helped explain how HIF-2α facilitated the proliferation and stemness of hPMSCs. However, NANOG and SOX2 exhibited a low transcription level and were unaffected by the changes in HIF-2α expression, which might be attributed to their distinct features in different types of stem cells. We examined significant signaling pathways to explore the signal transduction mechanisms that were regulated by HIF-2α and found that the components of the stress-responsive MAPK/ERK pathway were elevated in cells over-expressing HIF-2α. Conversely, the levels of these components were decreased in the HIF-2α-silenced hPMSCs compared with the negative control. We also found that VEGFA but not HIF-1α expression changed in response to changes in HIF-2α expression, suggesting that HIF-2α was the molecular regulator of the process described above. Therefore, HIF-2α was associated with the MAPK/ERK pathway at the level of mRNA expression. The main qRT-PCR results were checked using WB and IF to confirm that changes in gene expression were reflected in changes in the protein levels. These techniques verified that hPMSCs over-expressing HIF-2α exhibited increased levels of the active proteins CyclinD1, c-Myc, Oct4 and p-Erk1/2, and the HIF-2α-silenced hPMSCs expressed the lowest levels of these proteins. These results indicated that HIF-2α promoted the expression of CyclinD1 and c-Myc to facilitate hPMSC expansion. Similarly, Oct4 expression was elevated by HIF-2α and helped retain the original stem cell properties of hPMSCs. The regulation of Oct4 by HIF-2α may be a positive feedback mechanism to induce hPMSC proliferation. In the MAPK/ERK pathway, phosphorylated Erk1/2 represents the activation of the Ras-Raf-MEK-ERK cascade, which was connected to HIF-2α expression. PD0325901 was used to specifically inhibit MAPK/ERK signaling in hPMSCs to further investigate this process. The cellular proliferation and expression of CCND1 and MYC were markedly decreased in all inhibitor-treated groups compared with the control groups; in addition, HIF-2α silencing and inhibition of the MAPK/ERK pathway constituted the “dual suppression” function, which prevented the expression of proliferation-related genes in hPMSCs. Therefore, the inhibition of the MAPK/ERK pathway blocked hPMSC proliferation, which was facilitated by HIF-2α. In other words, HIF-2α might participate in MAPK/ERK signal transduction, thereby regulating hPMSC proliferation. MAPKs/ERKs are the downstream components of organized modules formed by scaffolding proteins and three kinases that sequentially activate each other by phosphorylation, among which, MAPK3 (human p44mapk, Erk1), the main effector, mediates cell proliferation upon activation. We applied EMSA to explore whether HIF-2α, as transcription factor, bound to and directly regulated the promoters of the signaling genes as a way to further analyze the concrete mechanism mediating the interaction of HIF-2α and the MAPK/ERK pathway. We analyzed the MAPK3 promoter sequence^23^ and found three putative hypoxia-response elements (HREs) – ACGTG^31^ – to which HIF-2α might bind and activate transcription. In the preliminary experiments, one potential binding site was selected and designed as a MAPK3 promoter probe. We found that hypoxic hPMSCs showed more HIF-2α–MAPK3 binding than the normoxic group, indicating that the transcription factor HIF-2α directly bound the “ACGTG” sequence in the MAPK3 promoter region in response to hypoxia. Therefore, HIF-2α regulated the transcription and activation of the MAPK3 gene, thereby promoting hPMSC proliferation. In addition, the ChIP-qPCR results also showed this intracellular binding and transcription.

**Figure 7:**
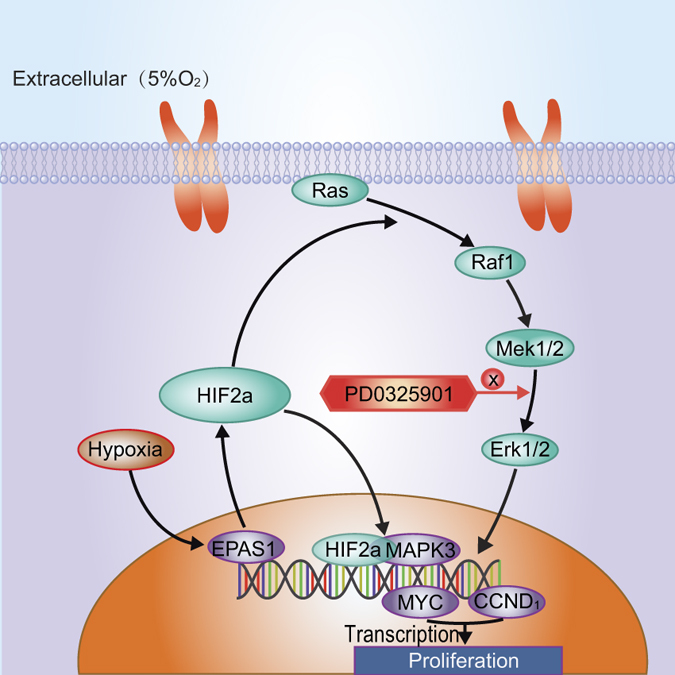
The proposed model for the regulatory mechanism of HIF-2α in hPMSCs through the MAPK/ERK pathway.

Taken together, the results indicated that HIF-2α stimulated the regenerative potential and maintained the original stemness of hPMSCs, which involved the action of CyclinD1 (CCND1), c-Myc (MYC) and Oct4 (POU5F1). Moreover, it was shown that HIF-2α directly and indirectly promoted hPMSC proliferation through the MAPK/ERK pathway. Based on the findings in this study, we propose a model for the regulatory mechanism of HIF-2α in hPMSCs (Fig. 7). Specifically, a physiologically hypoxic environment stimulates the transcription of HIF-2α, which also promotes hPMSC expansion. HIF-2α enhances the expression of the proliferation markers CyclinD1 and c-Myc, which improves the regenerative potential of hPMSCs. These results also explain the effects of hypoxia on hPMSCs because hypoxia initiates stress responses involved in cellular signal transduction. In general, the MAPK/ERK signaling pathway is activated by stress factors (e.g., low oxygen) and is also related to basic cellular activities (e.g., proliferation). HIF-2α over-expression, which mimics hypoxia, stimulates the Ras-Raf1-MEK1/2-ERK1/2 cascade, leading to the increased transcription of the proliferation related-factors CCND1 and MYC. Nevertheless, this function of HIF-2α can be prevented by the specific inhibitor PD0325901, which blocks the MAPK/ERK pathway. Furthermore, HIF-2α directly binds the MAPK3 promoter and activates its function through the interaction of the transcription factor and its regulated gene, which fundamentally explains how HIF-2α promotes hPMSC proliferation through the MAPK/ERK pathway in response to hypoxia.

This study provides a novel insight into the ability of HIF-2α to promote the regenerative potential of hPMSCs, as well as the mechanism involved, the MAPK/ERK pathway, which is crucial for clinical applications of hPMSCs and basic research. The “regulatory mechanism of HIF-2α in hPMSCs” model is supported by the results of our experiments. However, there may be other components of this complex mechanism that contribute to maintenance of the stem cell characteristics of hPMSCs, which will be examined in future experiments.

## Materials and Methods

### hPMSC cultures

hPMSCs were isolated as previously described^4^ and cultured in special medium (MesenCult^®^ Human Basal Medium plus MesenCult^®^ Human Supplement, STEMCELL Technologies Inc., Vancouver, Canada) in T25-cm^2^ cell culture flasks (Nunc™ EasYFlasks™, Thermo Fisher Scientific Inc., Waltham, MA, USA). First, the hPMSCs were cultured in a standard humidified incubator (HERAcell^®^150, Thermo Fisher Scientific Inc.) with 5% CO_2_ and atmospheric oxygen (21% O_2_) at 37 °C. During the continuous culture period, the culture medium was replaced every 3–4 days, and the cells were trypsinized and passaged upon reaching 70–80% confluence. After the hPMSCs were stabilized, the hypoxic group was moved to a particular humidified incubator (Forma™ Series II Water-Jacketed CO_2_ Incubators with Oxygen Control, Thermo Fisher Scientific Inc.) with 5% O_2_ and 5% CO_2_ at 37 °C for 4 hours, but the normoxic group remained in the original conditions. The oxygen concentration of the hypoxic group was explored and set in the pre-experiment. There were at least three independent samples in each group for every test.

### hPMSC infections

Once the cells reached 50% confluence, the hPMSCs were distributed into four groups for different treatments to acquire distinct levels of HIF-2α expression. Recombinant lentiviruses for HIF-2α (EPAS1) over-expression or the corresponding empty vectors (HA-HIF2alpha-pcDNA3, LV5-EF1a-GFP/Puro NC, Shanghai Gene Pharma Co., Ltd., Shanghai, China) were separately added to the medium to infect the respective groups of hPMSCs with an MOI of 100:1, with the aim of acquiring hPMSC that over-expressed HIF-2α and a corresponding negative control, hPMSCs-empty vector-NC. Similarly, two other groups of hPMSCs were individually infected with an HIF-2α shRNA for gene silencing (LV159-2-PL-shRNA-GFP-HOMO-EPAS1-1545, LV159-2-PL-shRNA-GFP-HOMO-EPAS1-417, Shanghai Novobio Technology Co., Ltd., Shanghai, China) or a scrambled shRNA for the negative control (LV159-GFP-NC, Shanghai Novobio Technology Co., Ltd.) with an MOI of 100:1 to acquire HIF-2α-silenced hPMSCs and a corresponding negative control, hPMSCs-scramble shRNA-NC. Polybrene (1:1000, Shanghai Gene Pharma Co., Ltd.) was mixed into every medium. The four groups of processed hPMSCs were cultured as usual for 48 hours, and the culture medium was replaced. Puromycin (Sigma-Aldrich Co. LLC, St. Louis, MO, USA) or blasticidin (Thermo Fisher Scientific Inc.) was mixed into each medium and filtered. After 2–3 days of extended culture, the medium was replaced and the filtering was repeated. Then, successful infection was confirmed by qRT-PCR, WBs, FACS assays (BD Accuri™ C6 Flow Cytometer, BD Biosciences, San Jose, USA), and confocal microscopy (Zeiss LSM710, Carl Zeiss AG, Oberkochen, Germany). Finally, the four groups of processed hPMSCs were continuously cultured. There were at least three independent samples in each group for every test.

As described above for the HIF-2α shRNA, initially, three pairs of shRNAs against HIF-2α (LV159-2-PL-shRNA-GFP-HOMO-EPAS1-1545, −2270, −417) (http://www.ncbi.nlm.nih.gov/gene/2034), and a scrambled shRNA (hPMSCs-scramble shRNA-NC), which was used as the negative control, were designed. The four sequences are presented in Supplementary Figure S2A. The qRT-PCR results showed that shRNA-1545, −2270, and −417 can all silence the expression of the HIF-2α mRNA, and shRNA-1545 had the best silencing effect, followed by shRNA-417. The WB results were the same as the qRT-PCR results (see Supplementary Figure S2B,C).

### Flow cytometry

Cell proliferation assays were conducted for each group of hPMSCs using Click-iT^®^ EdU Flow Cytometry Assay Kits (Thermo Fisher Scientific Inc.). According to the manufacturer’s instructions, EdU reagent was added to the different groups of hPMSCs in the culture medium (final concentration, 10 μM) to label the karyokinetic cells. Each group of assays was performed in triplicate. After a 2-hour incubation, the cells were washed with 1% bovine serum albumin (BSA, Sangon Biotech Corp., Shanghai, China) in phosphate-buffered saline (PBS, Hangzhou Gino Bio-pharmaceutical Technology Co. Ltd., Zhejiang, China) and pelleted by centrifugation. Click-iT^®^ fixative was mixed into the cell suspension at a ratio of 1 × 10^6 ^cells/100 μL in each tube and incubated for 15 minutes at room temperature in the dark. After washing with 1% BSA in PBS, the cells were incubated with the Click-iT^®^ saponin-based permeabilization and wash reagent for 15 minutes. A volume of 0.5 mL of Click-iT^®^ reaction cocktail was added to each tube and incubated for 30 minutes at room temperature in the dark. After washing once, the cell pellet was resuspended in 500 μL of Click-iT^®^ wash reagent. The negative control was processed in the same manner, except that the EdU reagent was omitted. Each assay was repeated three times independently. The cells in each group were analyzed using Beckman Coulter Cytomics FC 500 MPL (Beckman Coulter, Inc., Palo Alto, CA, USA).

The assay of the hPMSCs’ sizes depends on FSC/SSC analyses (forward scatter/side scatter) using a BD Accuri™ C6 Flow Cytometer (BD Biosciences, San Jose, CA, USA). The initial seeding density of hPMSCs was 5 × 10^4 ^cells/T75-cm^2^ cell culture flask. When the cells reached approximately 70% confluence, the hypoxic group was moved to a particular humidified incubator with 5% O_2_ and 5% CO_2_ at 37 °C for 24 hours, but the normoxic group was maintained in the original conditions. After routine washing, centrifugation and resuspension, the FSC/SSC of the hPMSCs were measured using FACS analyses. There were at least three independent samples in each group for every test.

Surface antigens analysis. The cells in each group were harvested and then washed with 0.5% BSA in PBS. The concentration of the cells in every tube was adjusted to 1 × 10^5 ^cells/100 μL. Next, the cells were separately incubated with APC-conjugated antibodies against human CD73, CD90, CD105, CD34 and CD45 (1:20, eBioscience Inc., San Diego, CA, USA) in the dark at room temperature for 30 minutes and then washed twice with PBS. Corresponding isotype antibodies were used as the controls to exclude non-specific binding. The experiments were performed in triplicate. The expression of surface antigens on the hPMSCs in each group was analyzed using a BD LSR II flow cytometer (Becton, Dickinson and Company, San Jose, CA, USA).

### Reverse transcription and quantitative real-time polymerase chain reaction analysis

Total RNA was extracted from the hPMSCs in each group using TRIzol reagent (Thermo Fisher Scientific Inc.) according to the manufacturer’s instructions. The quantity and quality of the isolated RNAs were evaluated by an ultraviolet (UV) spectrophotometer (SMA1000, Merinton Instrument Inc., Ann Arbor, MI, USA). The RNAs were reverse transcribed using PrimeScript™ RT Master Mix (TAKARA Biotechnology (Dalian) Co., Ltd., Liaoning, China) in an Eppendorf Mastercycler^®^ Nexus GX2 (Eppendorf AG, Hamburg, Germany), according to the manufacturer’s instructions. The cDNAs were used for qRT-PCR with TAKARA SYBR^®^ *Premix Ex Taq*™ II (TAKARA Biotechnology) and an Applied Biosystems^®^ 7500 Real-Time PCR System (Thermo Fisher Scientific Inc.). The primers were synthesized by Sangon Biotech Corp. and are shown in Supplementary Table S1. All qRT-PCR amplifications were performed in triplicate and repeated in three independent experiments. The relative differences in the expression levels of each selected gene were calculated (ΔΔCt) and reported as the fold induction (2^−ΔΔCt^). The relative mRNA quantities were normalized to glyceraldehyde 3 phosphate dehydrogenase (GAPDH), and the derived values of the experimental groups – hypoxic samples and hPMSCs with HIF-2α over-expression or silencing – were further normalized to the levels of the vehicle-treated groups: normoxic samples, or in other cases, the respective negative controls. One of the control samples was set to 1 in each condition.

### Western blot

The cells were harvested and lysed in RIPA lysis buffer (Beyotime Biotech Co., Ltd., Shanghai, China) containing protease inhibitor cocktail P8340 and phosphatase inhibitor cocktail P0044 (Sigma-Aldrich). The cells in solution were repeatedly vortexed until they were completely lysed and then centrifuged at 14,000 × *g* for 20 minutes. The supernatant was collected for the subsequent analysis. The protein concentration was determined using the Pierce™ BCA Protein Assay Kit (Thermo Fisher Scientific Inc.), according to the manufacturer’s instructions. All steps were performed on ice. Proteins (30 μg per sample) were electrophoresed on 10% Mini-PROTEAN^®^ TGX™ Gels (Bio-Rad Laboratories, Inc., Hercules, CA, USA) by SDS-PAGE in a Mini-PROTEAN^®^ Tetra Vertical Electrophoresis Cell (Bio-Rad Laboratories, Inc.) and transferred to PVDF membrane (Merck KGaA, Darmstadt, Germany) in a Mini Trans-Blot^®^ Cell (Bio-Rad Laboratories, Inc.). After incubation in blocking buffers containing BSA (Sangon Biotech Corp.) at room temperature for 1 hour, the membranes were incubated with specific primary antibodies (listed in Supplementary Table S2) overnight at 4 °C. Subsequently, the membranes were washed in Tris-buffered saline with 0.1% Tween (TBST, Sangon Biotech Corp.) three times and then incubated with horseradish peroxidase-conjugated donkey anti-mouse IgG or donkey anti-rabbit IgG (1:3,000, Sangon Biotech Corp.) for 1 hour at room temperature. After washing three times using TBST, the membranes were incubated with the Pierce™ ECL Western Blotting Substrate (Thermo Fisher Scientific Inc.) for 5–10 minutes. Finally, the signals of specific immunoreactive proteins were detected with the ChemiScope Western Blot Imaging System (Clinx Science Instruments Co., Ltd., Shanghai, China).

### Immunofluorescence microscopy

The cells were seeded into 4-well Millicell EZ SLIDE^®^ (Merck KGaA) plates and cultured until they were 70% confluent. After three washes with PBS, the cells were fixed with 4% formaldehyde (Wuhan Guge Biotech Ltd., Hubei, China) for 30 minutes in the dark and then washed three times with PBS and permeabilized with 0.1% Triton X-100 (Sangon Biotech Corp.) for 10 minutes. The cells were washed three times with PBS and blocked with 5% BSA in PBS for 1 hour. All steps were performed at room temperature. Then, the fixed cells were incubated with the specific primary antibodies listed in Supplementary Table S2 overnight in 4 °C, and the slides were incubated with goat anti-rabbit IgG (Alexa Fluor^®^ 647) (1:400, Abcam, Cambridge, UK) for 2 hours at room temperature in the dark. The cell nuclei were counterstained with DAPI (Beyotime Biotech Co., Ltd.) for 10 minutes. The slides were washed three times between each step of antibody incubation. The negative controls were processed in the same manner, except that the primary antibodies were omitted. The cells on the slides were observed and imaged using a confocal laser scanning microscope (Zeiss LSM710, Carl Zeiss AG, Germany).

### The inhibitor test

The growth of viable cells under the specified conditions (inhibitor or vehicle) was determined using a CCK-8 assay kit (Beyotime Biotech Co., Ltd.), according to the manufacturer’s instructions. Briefly, the hPMSCs in each group (5 × 10^3^ cells per well) were seeded in triplicate wells of Falcon^®^ 96-Well Cell Clear Flat Bottom Culture Plates (Corning Inc., Corning, NY, USA) and cultured for 24 hours. Then, the cells were treated with PD0325901 (100 μM in DMSO, Selleck Chemicals Inc., Houston, TX, USA) or an equivalent volume of DMSO (Sigma-Aldrich Co. LLC) for a total of 5 days. At every time point (every day), the medium in each well was replaced with 100 μL of fresh medium with 10 μL of CCK-8 reagent, and the plates were incubated for 2 hours at 37 °C. Afterwards, the absorbance was measured using a microplate spectrophotometer (Beckman Coulter DTX880 Multimode Detector, Beckman Coulter Inc.) at a wavelength of 450 nm. The cumulative optical density (OD) values of absorbance were plotted as a function of cell viability and proliferation in the inhibitor assay. From the second day, cell growth was determined by the difference in the absorbance on that day and the absorbance on the first day. In addition, the cells in the inhibitor-treated group were incubated with PD0325901 and the cells in the vehicle-treated group were incubated with DMSO for 2 days. The cell proliferation rate and gene expression levels in these groups were detected using the Click-iT^®^ EdU flow cytometry assay (Thermo Fisher Scientific Inc.) and qRT-PCR methods mentioned above.

### Electrophoretic mobility shift assay (EMSA)

Nuclear protein extracts were prepared from hPMSCs from the normoxic group or hypoxic group using cell nuclear and cytoplasmic extraction reagents (Beyotime Biotech Co., Ltd.) supplemented with protease and phosphatase inhibitors (Sigma-Aldrich Co. LLC.). Briefly, 2 × 10^6^ hPMSCs were harvested after washed with PBS. Then, 100 μL of cytoplasmic extraction reagent A was added to the cell pellet and briefly vortexed. After incubation on ice for 15 minutes, 10 μL of cytoplasmic extraction reagent B was mixed with the lysate and centrifuged at 14,000 × *g* for 5 minutes. The resulting supernatant was the cytoplasmic extract. Then, the insoluble fraction containing the nuclei was re-suspended with 50 μL of cell nuclear extraction reagent and placed on ice for 30 minutes with intermittent vortexing. The fraction was centrifuged at 14,000 × *g* for 10 minutes; the resulting supernatant was the nuclear extract, and the protein concentration of this extract was determined. EMSA was conducted using the lightshift chemiluminescent EMSA kit (Thermo Fisher Scientific Inc.), according to the manufacturer’s instructions. Eight micrograms (approximately 5 μL) of the nuclear protein extract were incubated with the probes. The sequences of double-stranded biotin-labeled MAPK3 probe1 (0.5 μL, 50 nM) was as follows:

5′-**Biotin**-CTCCCAAGTAGCTTGGATTACAGGC**ACGTG**CCACCATGCCCAGCTAATTT-3′

3′-GAGGGTTCATCGAACCTAATGTCCGTGCACGGTGGTACGGGTCGATTAAA-**Biotin**-5′.

The sequence of the unlabeled MAPK3 probe (0.5 μL, 10 μM) was as same as the above, without the biotin label on the 5′ end. All probes were annealed before use. The unlabeled MAPK3 probe was added as a competitor for the biotin-labeled probe. Eight micrograms of the anti-HIF-2α antibody (Abcam) were added to the supershift assay. The negative control was the biotin-labeled MAPK3 probe alone. Two microliters of 10× binding buffer, 1 μL of poly (dI·dC), 1 μL of 50% glycerol, 1 μL of 100 mM MgCl_2_, and 1 μL of 1% NP-40 were added to each reaction, and ultrapure water was added to bring the total volume to 20 μL. Then, the binding reactions were incubated at room temperature for 30 minutes. Five microliters of 5× loading buffer were added to each reaction, which was then loaded onto a 6% polyacrylamide gel with 0.5× TBE and separated at 100 V for 60 minutes. The gel was transferred to a Biodyne™ B Nylon membrane (Thermo Fisher Scientific Inc.) at 380 mA for 60 minutes in 0.5× TBE. The membrane was dried, crosslinked with UV light for 5 minutes, and dried on paper towels for 10 minutes. Twenty milliliters of pre-warmed 1× blocking buffer were added to the membrane for 15 minutes with gentle shaking, followed by a 15-minute incubation with 1:300-diluted streptavidin-horseradish peroxidase conjugate. The membrane was washed with 1× washing buffer, sequentially incubated with the substrate equilibration buffer and substrate working solution for 5 minutes, and then detected with the ChemiScope Chemiluminescent Imaging System (Clinx Science Instruments Co., Ltd.).

### Chromatin immunoprecipitation (ChIP) assay

The hPMSCs were grown to approximately 80% confluence in T175-cm^2^ cell culture flasks (Nunc™ EasYFlasks™) in a 5% or 21% oxygen environment for 4 hours, fixed with 1% paraformaldehyde (Guge Biotech Corp., Wuhan, China) diluted with 1× PBS, and quenched with 1.25 M glycine, pH 6.3 (Gibco). After sonication to yield DNA fragments of 300 to 500 base pairs, the chromatin was processed using the Millipore Chromatin Immunoprecipitation (ChIP) Assay Kit (Millipore, Billerica, MA, USA), according to the manufacturer’s protocol. The anti-HIF-2-alpha antibody-ChIP Grade (ab199, Abcam PLC, Cambridge, UK) was used for immunoprecipitation. The purified DNA was amplified by real-time PCR with an ABI 7500 instrument (Thermo Fisher Scientific Inc.) and TAKARA SYBR^®^ Premix Ex Taq™ II (TAKARA Biotechnology (Dalian) Co., Ltd.). The primers used to amplify the MAPK3 promoter were as follows: forward: 5′- AAGTGGTGCAATCTCAGCTCA-3′ and reverse, 5′- TGGGCAGATCACCTGAGTTT-3′. The fold enrichment was calculated using the formula 2^(−ΔCt [normalized ChIP^^] −^ ^ΔCt [normalized IgG])^. The entire experiment was repeated with 3 independent chromatin preparations.

### Image processing software packages

Images were cropped using Adobe Photoshop CS5 (ver: 12.0.3, Adobe Systems Software Ireland Ltd, CA, USA) and formatted using Adobe Illustrator CS5 (ver: 15.0.0, Adobe Systems Software Ireland Ltd, CA, USA).

### Statistical analyses

All tests were performed in triplicate and repeated in three independent experiments. The quantitative results are expressed as the mean ± standard deviations (S.D.). All data were analyzed to determine whether they were normally distributed. Differences between two groups were assessed by Student’s t-test, but one-way analysis of variance (ANOVA) was applied to compare multiple groups. Differences were considered significant at *p* < *0*.*05*. The data were analyzed using SPSS for Windows software (ver. 19.0; IBM Corp., NY, USA).

## Additional Information

**How to cite this article**: Zhu, C. *et al*. Hypoxia-inducible factor-2 alpha promotes the proliferation of human placenta-derived mesenchymal stem cells through the MAPK/ERK signaling pathway. *Sci. Rep.* **6**, 35489; doi: 10.1038/srep35489 (2016).

## Supplementary Material

Supplementary Information
