# Supplementary material for: Hypoxia-inducible factor-2 alpha promotes the proliferation of human placenta-derived mesenchymal stem cells through the MAPK/ERK signaling pathway
Source: Sci Rep. 2016 Oct 21;6:35489. doi: 10.1038/srep35489 (PMC5073233; doi:10.1038/srep35489)

Supplemental data

Hypoxia-inducible factor-2 alpha promotes the proliferation of human placenta-derived mesenchymal stem cells through the MAPK/ERK signaling pathway

Chengxing Zhu*, Jiong Yu*, Qiaoling Pan, Jinfeng Yang, Guangshu Hao, Yingjie Wang, Lanjuan Li, Hongcui Cao†

The State Key Laboratory for Diagnosis and Treatment of Infectious Diseases, First Affiliated Hospital, College of Medicine, Zhejiang University; Collaborative Innovation Center for Diagnosis and Treatment of Infectious Diseases,79 Qingchun Rd., Hangzhou City 310003, China

Correspondence to: Dr. Hongcui Cao, the State Key Laboratory for Diagnosis and Treatment of Infectious Diseases, First Affiliated Hospital, College of Medicine, Zhejiang University; Collaborative Innovation Center for Diagnosis and Treatment of Infectious Diseases,79 Qingchun Rd., Hangzhou City 310003, China; Email: hccao@zju.edu.cn

Table S1. The primers used for qRT-PCR

Gene/protein	Product size (bp)	Primer sequences
GAPDH	114	F: 5’-CTCTCTGCTCCTCCTGTTCG-3’<br>R: 5’-ACGACCAAATCCGTTGACTC-3’
EPAS1 (HIF-2α)	110	F: 5’- TGCTACGCCACCAGTACCA-3’<br>R: 5’- CAGTTCGGGCAGCAGGTAGG-3’
CCND1 (CyclinD1)	116	F: 5’- GCCCTCGGTGTCCTACTTCAAAT-3’<br>R: 5’-AGACCTCCTCCTCGCACTTCTGT-3’
MYC (c-Myc)	130	F: 5’- CCAGAGGAGGAACGAGCTAA-3’<br>R: 5’-TTGGACGGACAGGATGTATG-3’
POU5F1 (Oct4)	144	F: 5’- GGGAGATTGATAACTGGTGTGTT-3’<br>R: 5’-GTGTATATCCCAGGGTGATCCTC-3’
NANOG	116	F: 5’-TTTGTGGGCCTGAAGAAAACT-3’<br>R: 5’- AGGGCTGTCCTGAATAAGCAG-3’
SOX2	110	F: 5’- TACAGCATGTCCTACTCGCAG-3’<br>R: 5’-GAGGAAGAGGTAACCACAGGG-3’
KRAS	119	F: 5’-CACGGTCATCCAGTGTTGTC-3’<br>R: 5’-GCTCTTGATTTGTCAGCAGGA-3’
RAF1	122	F: 5’-CCTCCAGTCCCTCATCTGAA-3’<br>R: 5’-TCGAATTGCATCCTCAATCA-3’
MAP2K1 (Mek1)	113	F: 5’-GCTGTTCCTGCTCCATGACT-3’<br>R: 5’- TCTCACAAGGCTCCCTCCTA-3’
MAP2K2 (Mek2)	135	F: 5’-CCAAGGTCGGCGAACTCAAA-3’<br>R: 5’-TCTCAAGGTGGATCAGCTTCC-3’
MAPK3 (Erk1)	123	F: 5’-CTGGCAAGCACTACCTGGAT-3’<br>R: 5’-TGGAGGGCAGAGACTGTAGG-3’
MAPK1 (Erk2)	169	F: 5’- TACACCAACCTCTCGTACATCG-3’<br>R: 5’-CATGTCTGAAGCGCAGTAAGATT-3’
VEGFA	115	F: 5’-GGTCCCTCTTGGAATTGGAT-3’<br>R: 5’- TGTATGTGGGTGGGTGTGTC-3’
HIF1A (HIF-1α)	118	F: 5’-TGCAACATGGAAGGTATTGC-3’<br>R: 5’- TTCACAAATCAGCACCAAGC-3’

Table S2. Primary antibodies used for western blot and immunofluorescence

Primary antibodies/ product codes	Applications/dilutions	Company
Anti-GAPDH mouse monoclonal antibody (D190090)	WB 1:4000 (36 kDa)	Sangon
Anti-HIF-2-alpha rabbit polyclonal antibody (ab199)	WB 1:500 (115 kDa); IF 1:100	Abcam
Anti-Oct4 rabbit polyclonal antibody (ab19857)	WB 1:200 (50 kDa); IF 1:100	Abcam
Anti-Cyclin D1 rabbit monoclonal antibody (ab134175)	WB 1:3000 (34 kDa); IF 1:100	Abcam
Anti-c-Myc rabbit monoclonal antibody (5605s)	WB 1:1000 (60 kDa); IF 1:200	CST
Anti-Erk1(pT202/pY204) + Erk2(pT185/pY187) rabbit monoclonal antibody (ab32538)	WB 1:700 (42kDa,44kDa); IF 1:200	Abcam

Figure S1:

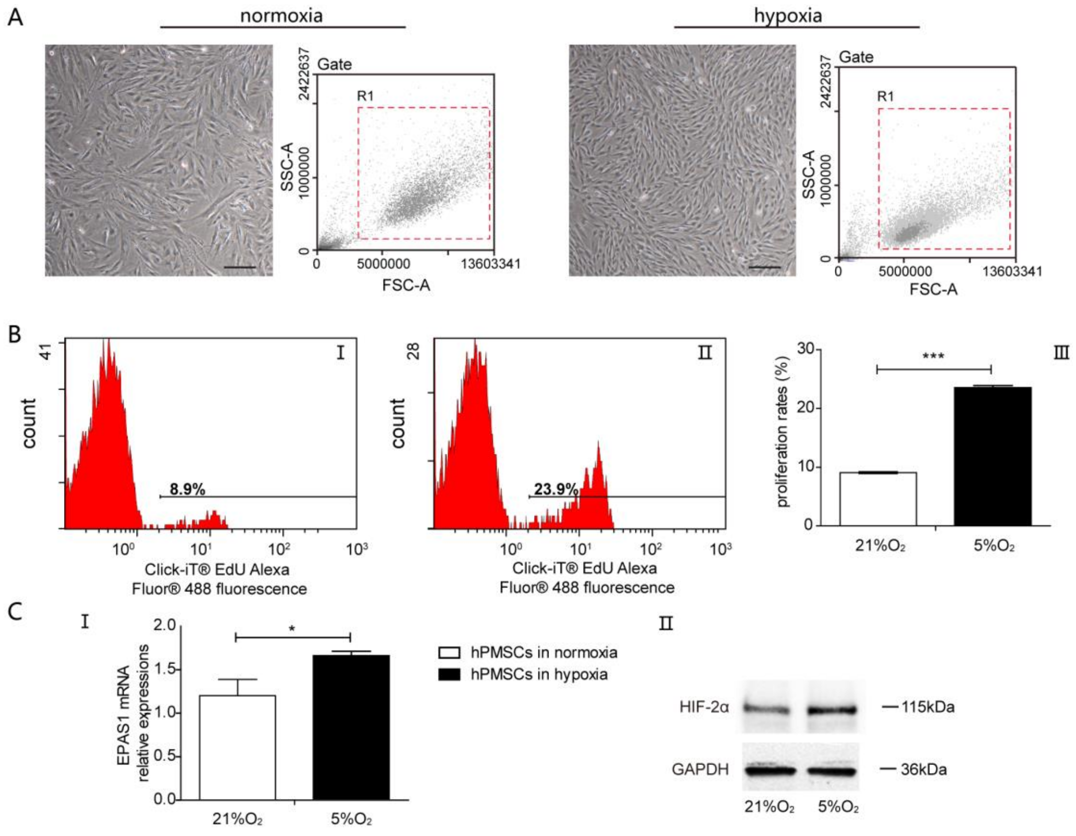

Comparison of the general biological characteristics between normoxic and hypoxic human placenta-derived mesenchymal stem cells (hPMSCs).

A: Morphologies (4×, scale bars, 200 μm) and sizes (FSC *vs*. SSC assays) of the hPMSCs: (I) hPMSCs cultured under normoxic conditions and (II) hPMSCs cultured under hypoxic conditions. The results showed that hPMSCs became comparatively smaller in a hypoxic atmosphere. FSC, forward scatter; SSC, side scatter.

B: EdU flow cytometry cell proliferation rates: (I) proliferation rate of normoxic hPMSCs, (II) proliferation rate of hypoxic hPMSCs, and (III) statistical graph of the different proliferation rates. The tests were performed in triplicate and repeated in three independent experiments. The data are presented as the mean ± S.D. (error bars) and were statistically analyzed using Student's t-test. *** $p < 0.001$.

C: Comparison of the relative levels of HIF-2α mRNA and protein between the normoxic and hypoxic hPMSCs. (I): qRT-PCR result. The qRT-PCR tests were performed in triplicate and repeated in three independent experiments. The data are presented as the mean ± S.D. (error bars) and were statistically analyzed using Student's t-test. * $p < 0.05$. (II): Western blot result. Lane 1: Level of the HIF-2α protein in normoxic hPMSCs (21% O_2); Lane 2: Level of the HIF-2α protein in the hypoxic hPMSCs (5% O_2). The HIF-2α protein is approximately 115 kDa. The WB result shows that HIF-2α protein level in the hypoxic group is slightly increased compared with the normoxic group.

Figure S2:

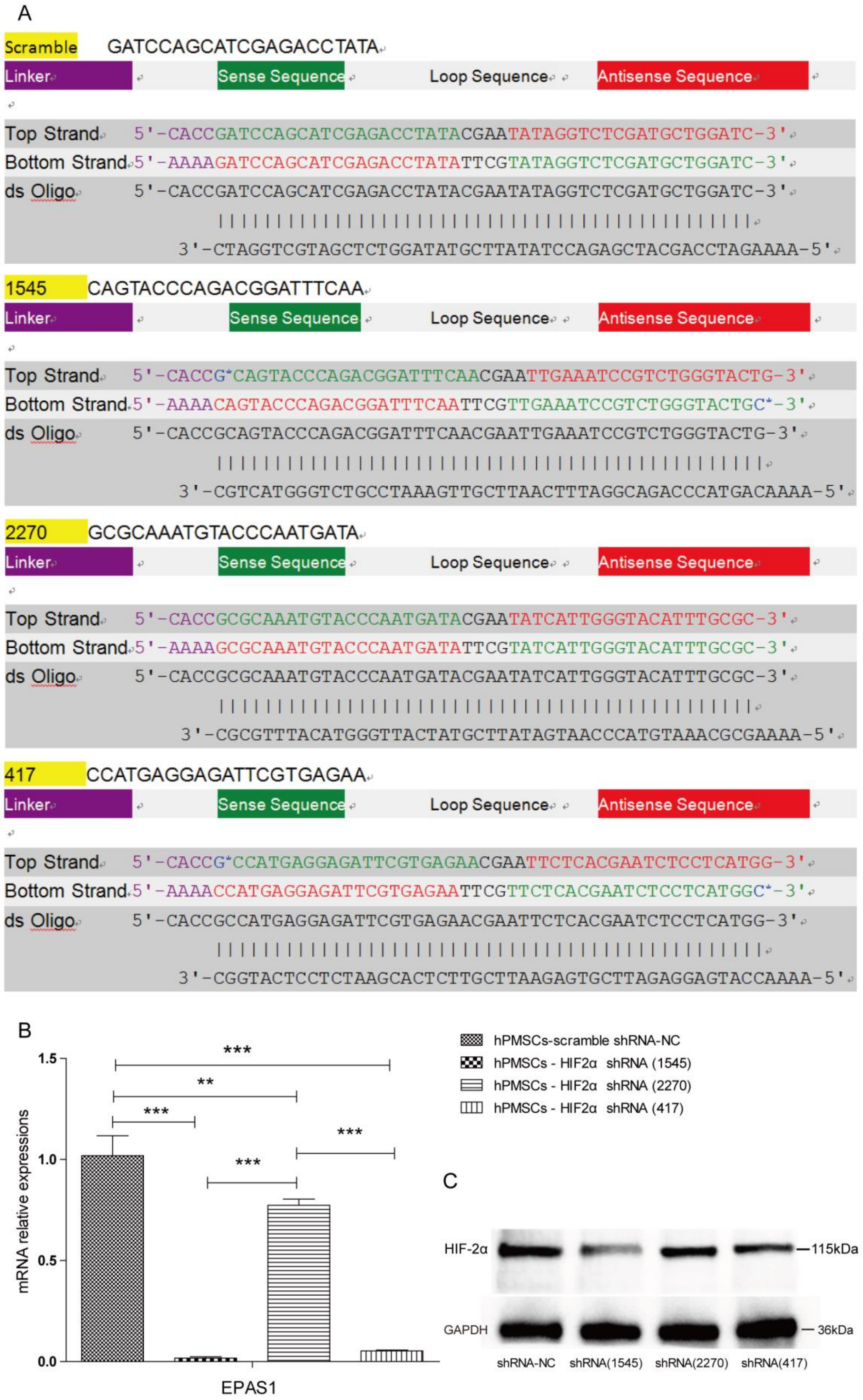
A
Scramble GATCCAGCATCGAGACCTATA
Linker
Sense Sequence
Loop Sequence
Antisense Sequence
Top Strand 5'-CACCGATCCAGCATCGAGACCTATACGAATATAGGTCTCGATGCTGGATC-3'
Bottom Strand 5'-AAAAGATCCAGCATCGAGACCTATATTCGTATAGGTCTCGATGCTGGATC-3'
ds Oligo 5'-CACCGATCCAGCATCGAGACCTATACGAATATAGGTCTCGATGCTGGATC-3'
3'-CTAGGTCGTAGCTCTGGATATGCTTATATCCAGAGCTACGACCTAGAAAA-5'
1545 CAGTACCCAGACGGATTTCAA
Linker
Sense Sequence
Loop Sequence
Antisense Sequence
Top Strand 5'-CACCG*CAGTACCCAGACGGATTTCAACGAATTGAAATCCGTCTGGGTACTG-3'
Bottom Strand 5'-AAAACAGTACCCAGACGGATTTCAATTCGTTGAAATCCGTCTGGGTACTGC*-3'
ds Oligo 5'-CACCGCAGTACCCAGACGGATTTCAACGAATTGAAATCCGTCTGGGTACTG-3'
3'-CGTCATGGGTCTGCCTAAAGTTGCTTAACTTTAGGCAGACCCATGACAAAA-5'
2270 GCGCAAATGTACCCAATGATA
Linker
Sense Sequence
Loop Sequence
Antisense Sequence
Top Strand 5'-CACCGCGCAAATGTACCCAATGATACGAATATCATTGGGTACATTTGCGC-3'
Bottom Strand 5'-AAAAGCGCAAATGTACCCAATGATATTCGTATCATTGGGTACATTTGCGC-3'
ds Oligo 5'-CACCGCGCAAATGTACCCAATGATACGAATATCATTGGGTACATTTGCGC-3'
3'-CGCGTTTACATGGGTTACTATGCTTATAGTAACCCATGTAAACGCGAAAA-5'
417 CCATGAGGAGATTCGTGAGAA
Linker
Sense Sequence
Loop Sequence
Antisense Sequence
Top Strand 5'-CACCG*CCATGAGGAGATTCGTGAGAACGAATTCTCACGAATCTCCTCATGG-3'
Bottom Strand 5'-AAAACCATGAGGAGATTCGTGAGAATTCGTTCTCACGAATCTCCTCATGGC*-3'
ds Oligo 5'-CACCGCCATGAGGAGATTCGTGAGAACGAATTCTCACGAATCTCCTCATGG-3'
3'-CGGTACTCCTCTAAGCACTCTTGCTTAAGAGTGCTTAGAGGAGTACCAAAA-5'
B
mRNA relative expressions
1.5
1.0
0.5
0.0
EPAS1
***
**
***
***
***
hPMSCs-scramble shRNA-NC
hPMSCs - HIF2α shRNA (1545)
hPMSCs - HIF2α shRNA (2270)
hPMSCs - HIF2α shRNA (417)
C
HIF-2α
GAPDH
115kDa
36kDa
shRNA-NC
shRNA(1545)
shRNA(2270)
shRNA(417)

Four shRNA sequences aimed at silencing the HIF-2α gene silencing (A) and their silencing effects (B-C).

A: shRNA sequences (1545, 2270, 417) and a negative control group (scramble shRNA-NC).

B: The qPCR results shows that shRNA (1545, 2270,417) can silence the mRNA relative expression of HIF-2α, and shRNA-1545 has the best silencing effect, followed by shRNA 2270.

C: The western blot results are presented as follows (from left to right): hPMSCs-scramble shRNA-NC, hPMSCs - HIF2α shRNA-1545, hPMSCs - HIF2α shRNA-2270, hPMSCs - HIF2α shRNA-417. The results showed that shRNA-1545 had the best silencing effect.

Figure S3: The complete original immunoblots shown in Figure 3 are presented in order below. The individual parts comprising Figure 3 are specified using red boxes.

CyclinD1

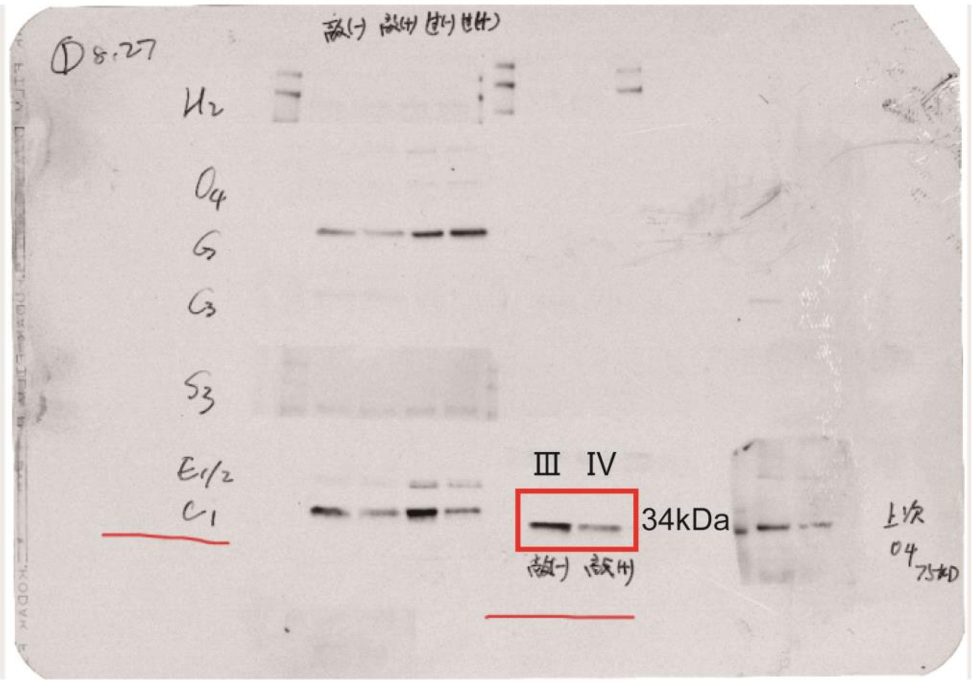

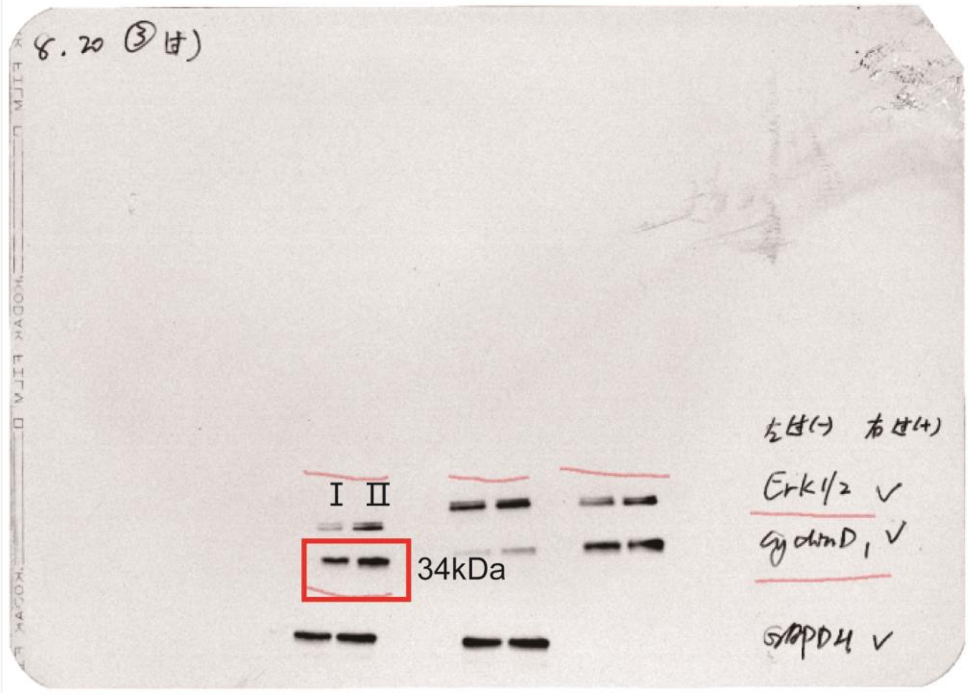

GAPDH

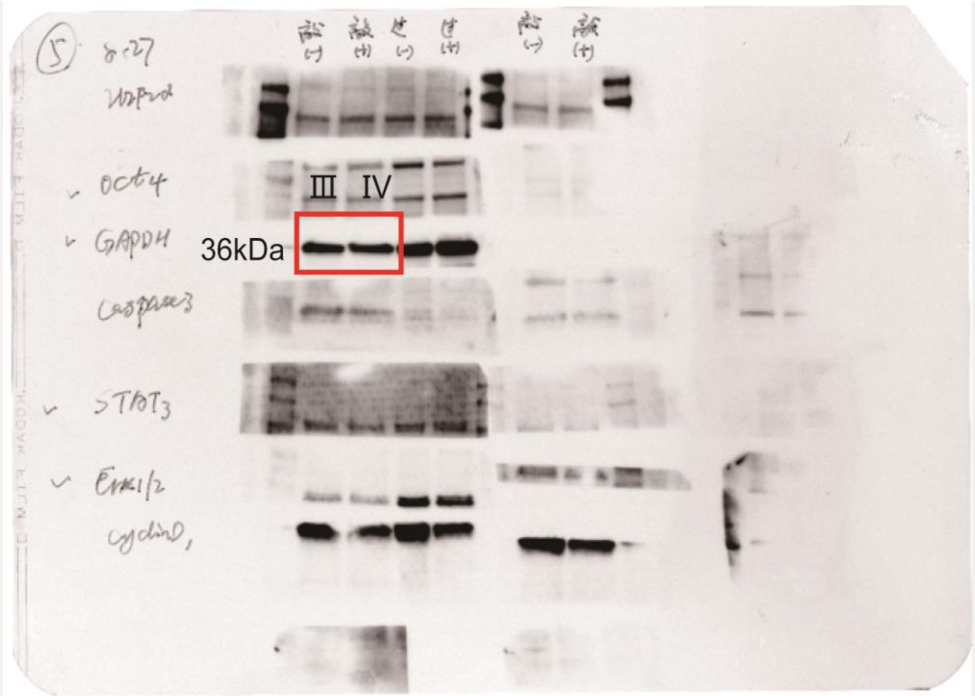

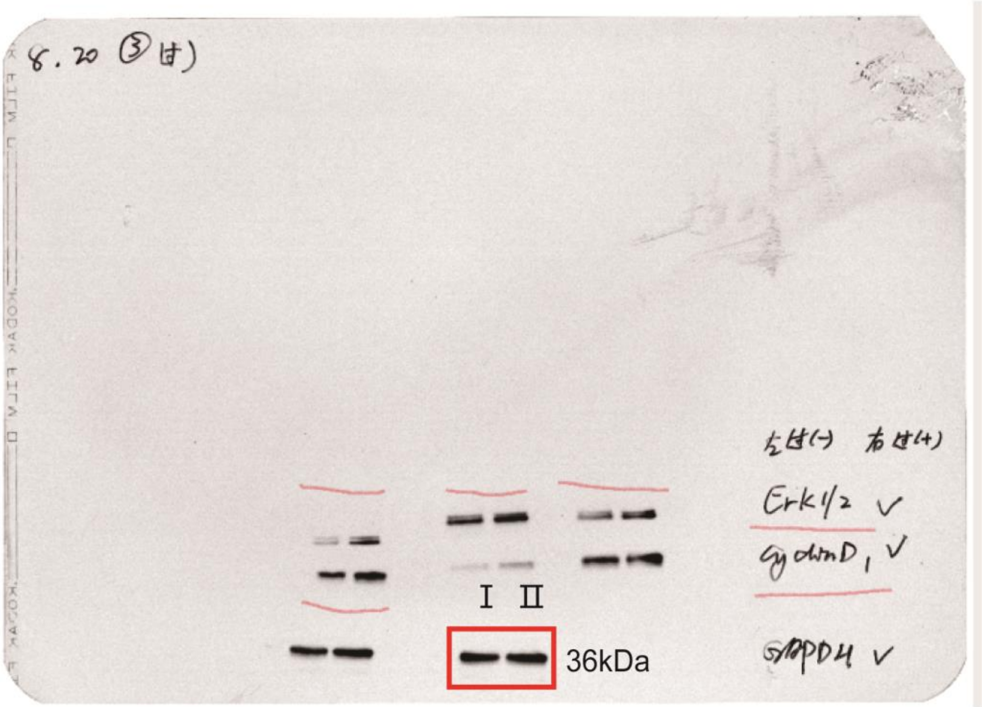

HIF-2α

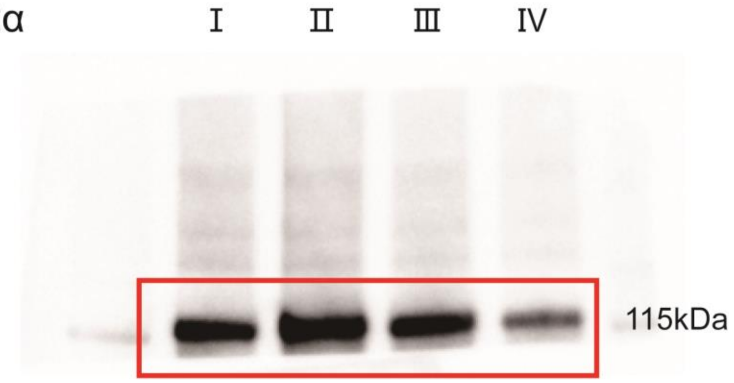

c-Myc

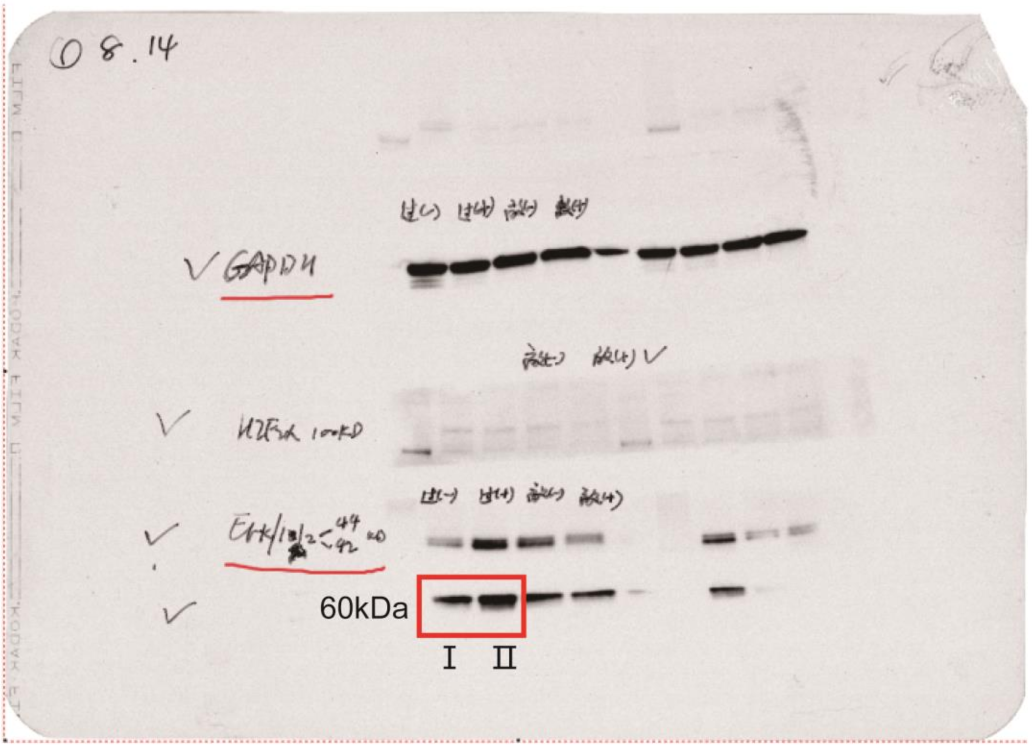

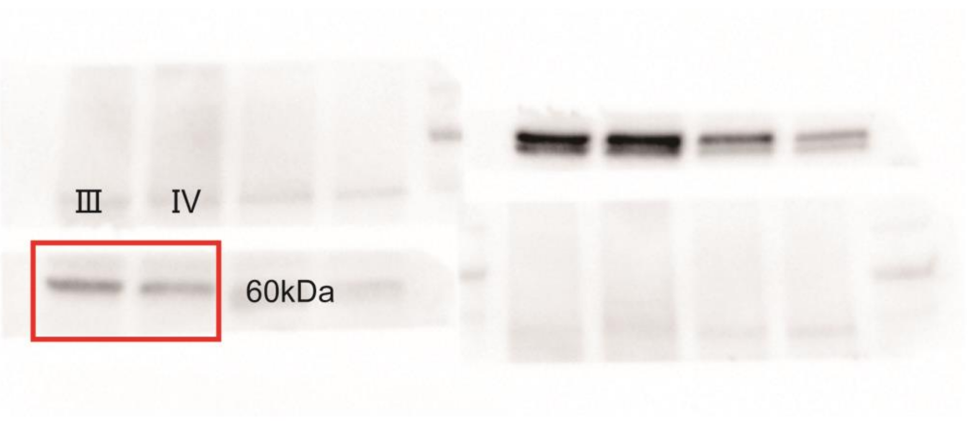

Oct4

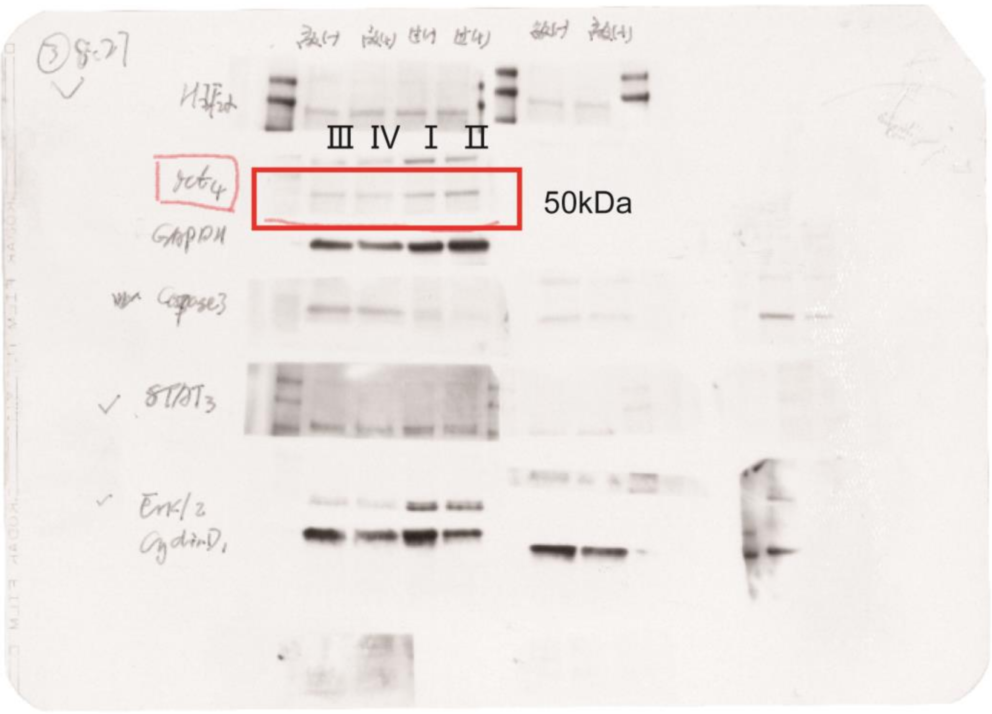

p-Erk1/2

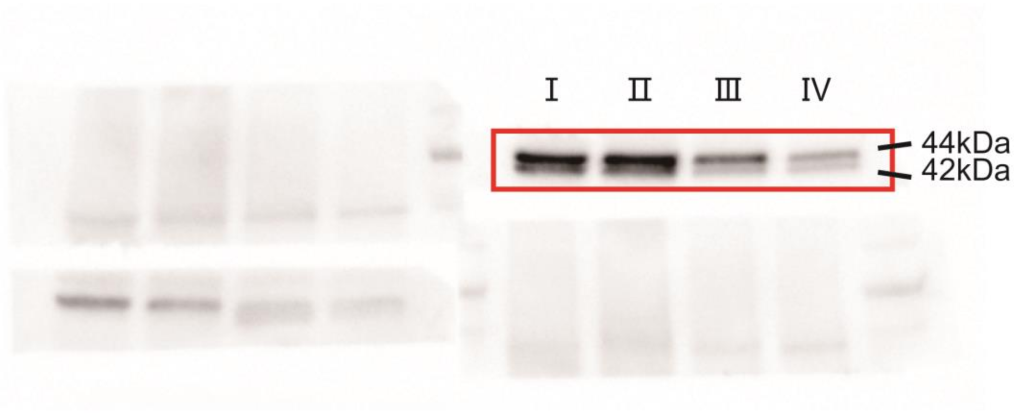

Supplement: Supplementary Information [file srep35489-s1.pdf]
